# Surveillance for Prion Disease in Cervids, Germany

**DOI:** 10.3201/eid1202.050970

**Published:** 2006-02

**Authors:** Elvira Schettler, Falko Steinbach, Iris Eschenbacher-Kaps, Kirsten Gerst, Franz Meussdoerffer, Kirsten Risch, Wolf Jürgen Streich, Kai Frölich

**Affiliations:** * Institute for Zoo and Wildlife Research, Berlin, Germany;; † Veterinary Laboratories Agency, Weybridge, United Kingdom;; ‡ Cenas AG, Kulmbach, Germany;; § Landesveterinär- und Lebensmitteluntersuchungsamt Mecklenburg-Vorpommern, Rostock, Germany;; ¶ University of Bayreuth, Bayreuth, Germany

**Keywords:** Transmissible spongiform encephalopathy (TSE), prion disease, chronic wasting disease (CWD), Europe, Germany, risk analysis, screening, cervids, fallow deer, roe deer, red deer

## Abstract

An active survey on transmissible spongiform encephalopathies was performed from 2002 to 2005 on 4,255 roe deer, 1,445 red deer, and 1,604 fallow deer in Germany. All cervids tested negative. This survey has been the largest in European wildlife and provides no evidence of prion diseases in free-living German cervids.

Germany has one of the largest deer populations in Europe (*1*), and ≈19,000 tons of venison are consumed in Germany each year (*2**,**3*). In light of the increasing number of scrapie cases, presence of bovine spongiform encephalopathy (BSE) in Germany (*4*), and increasing prevalence of chronic wasting disease (CWD) in North America, concern exists that transmissible spongiform encephalopathies (TSE) could also affect German wildlife, especially cervids. TSE could be transmitted to German cervids through importation of infected cervids; by their sharing habitats with other infected animals (scrapie from sheep); by exposure to BSE-contaminated meat, bone meal, or milk powder; or by exposure to other European cervids (spontaneous form of TSE). Until now, TSE has not been shown to exist in European deer. However, little surveillance has taken place (*1*). In Germany, only passive surveillance on suspected deer and a TSE survey that focused exclusively on Bavarian cervids have been conducted (*5*). However, risk for human exposure cannot be excluded until sufficient surveillance has been performed (*6*).

## The Study

The objective of our study was to determine possible TSE occurrence in cervids from Germany from 2002 to 2005. Our target species were the 3 main cervid species, roe deer (*Capreolus capreolus*), red deer (*Cervus elaphus elaphus*), and fallow deer (*Dama dama*). Since TSE in young animals is unlikely (*7*), only adult animals (age >18 months) were studied. Information on distribution of age groups was obtained from local hunting authorities. The target population of our 3-year study was ≈3,492,000 roe deer, 181,000 red deer, and 157,000 fallow deer (Table 1). The population size was estimated by assuming that the annual hunting bag (number of animals killed each year) represents approximately one third of the population, that age distributions in the hunting bag correspond to those of the deer population, and that the annual population sizes before hunting did not change during the study period. These assumptions correspond to management regulations for hunting (*2*). During the 2002–2003 hunting season, 1,117511 roe deer, 60,407 red deer, and 52,240 fallow deer were killed in Germany (*2*). On the basis of these data, the hunting bags of cervids >18 months for the 3-year study period were estimated at 2,095,000 roe deer, 109,000 red deer, and 94,000 fallow deer (Table 1).

**Table 1:** Minimum prevalence levels evaluated for German cervids tested for TSE, 2002–2005*

Species	Increased risk†			Normal risk‡			Total§		
	No. tested (MPL)	HB	EPS	No. tested (MPL)	HB	EPS	No. tested	HB	EPS
Roe deer	1,959 (0.15%)	822,000	1,370,000	1,684 (0.18%)	1,273,000	2,122,000	3,643	2,095,000	3,492,000
Red deer	1,110 (0.27%)	84,000	140,000	297 (1.00%)	25,000	42,000	1,407	109,000	181,000
Fallow deer	1,097 (0.27%)	76,000	127,000	293 (1.02%)	18,000	30,000	1,390	94,000	157,000

*TSE, transmissible spongiform encephalopathy; MPL, minimum prevalence level (upper limit of the percentage of positives in the population, given no positives found in the sample); HB, hunting bag (cervids >18 months estimated for the 3 study years); EPS, estimated population size (cervids >18 months in the 3 study years). †Animals were considered at increased risk if >1 risk factor applied. ‡Animals were considered at normal risk if no risk factors applied. §Data analysis was possible for 6,440 animals; information on risk factors was lacking in 616 cases.

The target region was all of Germany; the 323 administrative districts of Germany were our sampling areas (Figure). Within these districts, samples were taken from different hunting areas to ensure including as many local deer populations as possible. However, the hunting areas are not necessarily identical to home ranges of deer populations. As CWD in North America tends to occur focally, this strategy was chosen to ensure that potential foci would not be missed.

**Figure:**
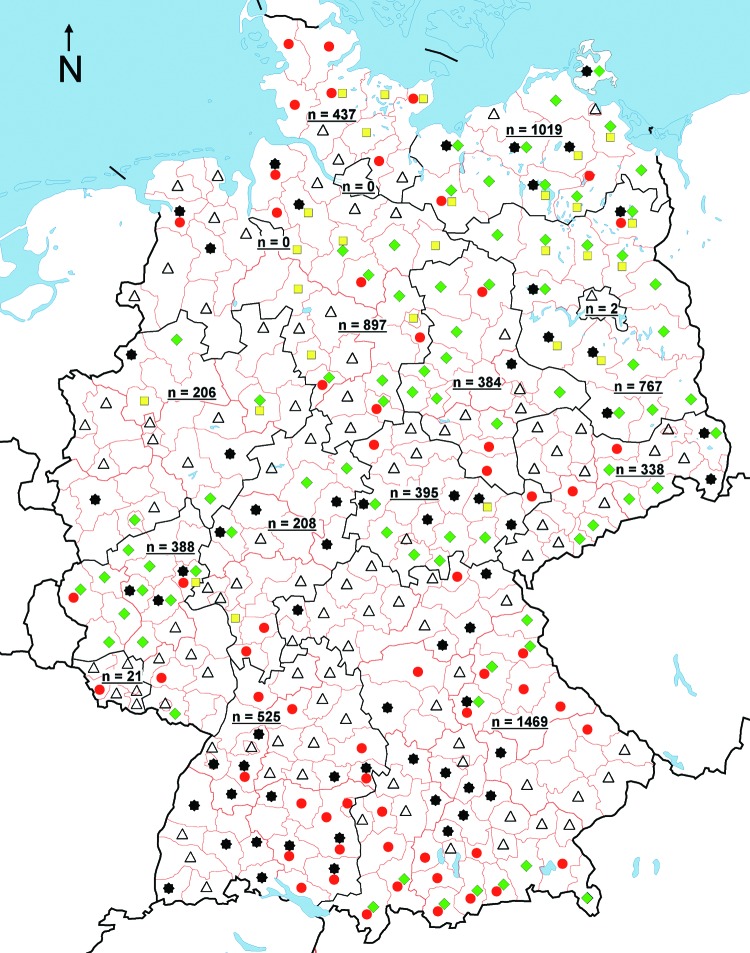
Distribution of free-ranging roe deer, red deer, and fallow deer tested for transmissible spongiform encephalopathies that shows the risk for each district where samples were obtained. 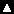, samples originating from a district without any risk attributes; 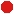, samples originating from a district where BSE incidence in cattle was higher than average BSE incidence in Germany; 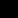, samples originating from a district with occurrence of scrapie in domestic sheep; 

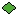
, samples from red deer originating from a district with high red deer density; 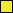, fallow deer samples originating from a district with high fallow deer density; n, number of samples from each federal state. Samples came from 14 (88%) of the 16 federal states (2 missing states are 2 major cities with almost no deer population) and from 280 (87%) of the 323 German administrative districts.

Several attributes may be associated with greater probability of TSE (*8*). Accordingly, we defined 2 risk categories for each species, an increased risk and a normal risk category. Animals were considered to be at increased risk for TSE if >1 of the following conditions applied: 1) BSE incidence in cattle (also relating to their parentage) in the district was higher than the average BSE incidence in Germany, 2) scrapie occurred in sheep in the district, or 3) fallow deer or red deer were distributed in districts with high density. If the disease were endemic, this method would be more likely to detect it in these areas. Moreover, special sampling efforts were directed toward animals that showed cachexia and central nervous system disorders and animals that were found dead.

Because we expected low prevalence or lack of TSE in our target population, we expected to find any positive animals only in the increased risk category. We wanted to ensure that our sample sizes were adequate to detect TSE even at a prevalence of 0.5% for cervids at increased risk and 1% for cervids at normal risk with 95% confidence. The respective sample sizes were calculated by using the approximation formula of the hypergeometric distribution (*9*). We than stratified the sample by hunting bag of each district.

Data analyses were performed on the basis of the hunting bag as well as on the estimated population of cervids >18 months of age in the 3-year study period (Table 1). For each sample, we retrospectively calculated the minimum prevalence level (MPL). MPL is an upper limit to the percentage of positives in the population if no positives are found in the sample (*9*). It represents the detection threshold below which the survey cannot detect a TSE infection at the 95% confidence level. Data from captive cervids were regarded as 1 sample and analyzed separately.

Foresters, hunters, and game farmers submitted the heads of 7,056 free-living and 248 captive deer that had been hunted, found dead, or suspected of having disease. Samples from free-living deer were obtained from 280 (87%) of the 323 German districts. Samples from captive deer originated from 43 of ≈6,000 German deer farms and from 12 of 16 federal states. Data from collected deer included species, age (estimated on tooth patterns), sex, location of kill, and health status (Table 2). Brain stem (obex region) and medial retropharyngeal lymph nodes were tested for TSE by Platelia BSE enzyme-linked immunosorbent assay (Bio-Rad Laboratories GmbH, Munich, Germany) according to manufacturer’s instructions (*1**,**11*). Recombinant bovine prion protein was the positive control.

**Table 2:** Free-living German cervids tested for transmissible spongiform encephalopathies, 2002–2005

Characteristic	Roe deer	Red deer	Fallow deer	Total
No. animals tested	4,250	1,416	1,390	7,056
Sex				
Female	3,137	1,257	1,246	5,640
Male*	502	148	144	794
Unknown	611	11	0	622
Age (y)				
<2	279	127	154	560
2–3	2,196	743	841	3,780
4–6	890	383	329	1,602
>6	273	154	66	493
Unknown	612	9	0	621
Increased risk category	1,959	1,110	1,097	4,166
BSE risk†	1,409	334	236	1,979
Scrapie risk‡	693	215	443	1,351
Fallow deer high density§	–	–	1,035	1,035
Red deer high density§	–	1,030	–	1,030
Clinical suspects¶	55	9	5	69
Found dead	123	16	3	142
Normal risk category#	1,684	297	293	2,274
No BSE risk	2,234	1,073	1,154	4,461
No scrapie risk	2,950	1,192	947	5,089
Fallow deer low density	–	–	355	355
Red deer low density	–	377	–	377
Cervids with unknown risk**	607	9	0	616

*Relatively few male animals were tested. According to a recent report, male cervids appear to be at higher risk for chronic wasting disease than sympatric females (*10*). †Animal came from a district where bovine spongiform encephalopathy (BSE) incidence in cattle was higher than the average BSE incidence in Germany (true for all target species). ‡Animal came from a district with occurrence of scrapie in sheep (true for all target species). §Fallow deer distributed in districts with high fallow deer density or red deer distributed in districts with high red deer density. ¶Cachexia and central nervous system disorders. #An animal was allocated to the normal risk category if none of the above risk factors applied. **Animals (n = 616) with unknown risk attributes were not included in risk analysis.

Protease-resistant prion protein (PrP^res^) was not detected in any samples from free-living roe deer (n = 4,250), red deer (n = 1,416), or fallow deer (n = 1,390). Regarding the different risk categories, data analysis was possible for 6,440 animals (Table 1). MPLs for the 3-year populations differed by no more than 0.001% from those calculated for the hunting bags. More than 200 samples came from animals with suspected disease or animals found dead. All 248 captive cervids were negative for TSE (Table 2). Because of the small sample size, MPLs of 1.39% for fallow deer (n = 214) and 9.81% for red deer (n = 29) were accordingly high.

## Conclusions

This study represents the largest surveillance program on TSE in European wildlife. Eighty-seven percent of all German districts were covered by our investigation (Figure). Recent data show that CWD prevalence in mule deer varied, depending on whether samples had been collected in biologically relevant units or in administrative jurisdictions (*10*). For logistic reasons, our sampling areas did not always completely cover biologically relevant geographic units. However, we ensured that within each district, samples were derived from different hunting areas so that potential TSE foci would not be missed.

Moreover, our findings were below the targeted minimum prevalence levels of 0.5% for cervids at increased risk (Table 1). The overall CWD prevalence observed in free-living cervids in disease-endemic areas of Colorado and Wyoming is ≈5% for mule deer (*Odocoileus hemionus*), 2% for white-tailed deer (*O. virginianus*), and <1% for elk (*Cervus elaphus nelsoni*) (*7*). In Wisconsin, where CWD only occurs focally, an overall prevalence of 0.62% was detected in free-living white-tailed deer (*12*). In our study, we reached lower detection limits.

If CWD or any other TSE were present in our target population in Germany at a minimum prevalence of 0.15% to 1.02%, depending on the species and risk category analyzed, we should have detected >1 infected animal with a 95% probability (*8*). As no PrP^res^ was detected, our study does not indicate that TSE is present in free-living cervids in Germany. Even if TSE occurs in German cervids, it is not widely distributed. The negative results seen with captive cervids in our study are of limited significance, since the sample size was small. Here, more risk analysis is required.

Apart from surveillance, more experimental research on transmission of TSE is required; we have not resolved whether European deer species are susceptible to CWD or other TSE (*1*). As with all prion diseases, a species barrier seems to exist for CWD (*1*); moose (*Alces alces*) and caribou (*Rangifer tarandus*) have not been found naturally infected with disease, even in CWD-endemic areas (*1**,**13*). A higher risk for CWD can be assumed for red deer since they belong to the same species as elk (*1*). BSE was only observed in different species from the families *Bovidae* and *Felidae* in zoos (*14*) but not in members of the family *Cervidae*, even though they were most likely also exposed to BSE-contaminated food (*6*). Our study indicates that TSE is unlikely to exist in free-living cervids from Germany and that the risk for TSE transmission to humans from eating venison is low.
